# Association of changes in cardiorespiratory fitness with health-related quality of life in young adults with mobility disability: secondary analysis of a randomized controlled trial of mobile app versus supervised training

**DOI:** 10.1186/s12889-020-09830-y

**Published:** 2020-11-16

**Authors:** Anna-Maria Lampousi, Daniel Berglind, Yvonne Forsell

**Affiliations:** Department of Global Public Health, Karolinska Institutet, Stockholm, Sweden

**Keywords:** Cardiorespiratory fitness, Mobile app, Supervised training, Mobility disability, Health related quality of life, HRQoL, Young adults

## Abstract

**Background:**

Young adults with mobility disability report lower health-related quality of life (HRQoL) than their able-bodied peers. This study aims to examine potential differences between the effects of mobile app versus supervised training and the association of cardiorespiratory fitness change with HRQoL in young adults with mobility disability.

**Methods:**

This is a secondary analysis of a parallel randomized controlled trial of a mobile app (*n* = 55) and a supervised health program (*n* = 55) that was provided for 12 weeks to 110 adults (18–45 years) with self-perceived mobility disability. Recruitment took place at rehabilitation centers in Stockholm, Sweden. Cardiorespiratory fitness was estimated from the results of a submaximal cycle ergometer test and HRQoL was assessed with the SF-36 questionnaire. Follow up was at 6 weeks, 12 weeks, and 1-year and all examinations were performed by blinded investigators. Between group differences of changes in HRQoL at follow up were estimated in intention-to-treat analysis using linear regression models. Crude and adjusted mixed-effects models estimated the associations between cardiorespiratory fitness change and HRQoL. Stratified analysis by intervention group was also performed.

**Results:**

In total, 40/55 from the mobile app group and 49/55 from the supervised training group were included in the intention to treat analysis. No significant differences were observed between the effects of the two interventions on HRQoL. In both crude and adjusted models, cardiorespiratory fitness change was associated with the general health (adjusted β = 1.30, 95% CI: 0.48, 2.13) and emotional role functioning (adjusted β = 1.18, 95% CI: 0.11, 2.25) domains of SF-36. After stratification, the associations with general health (adjusted β = 1.88, 95% CI: 0.87, 2.90) and emotional role functioning (adjusted β = 1.37, 95% CI: 0.18, 2.57) were present only in the supervised group.

**Conclusion:**

This study found positive associations between cardiorespiratory fitness change and HRQoL in young adults with mobility disability who received supervised training. The effects of mobile app versus supervised training on HRQoL remain unclear.

**Trial registration:**

International Standard Randomized Controlled Trial Number (ISRCTN) registry ISRCTN22387524; Prospectively registered on February 4th, 2018.

## Background

Disability is a condition that any person might encounter at some point in life as a consequence of health conditions [1]. It can be experienced differently by each individual depending on interactions between medical and contextual factors, as indicated in the International Classification of Functioning, Disability and Health (ICF) model developed by the World Health Organization (WHO) [2]. Mobility disability is the most common form of disability among Swedish adults, affecting approximately 13% of males and 19% of females [3]. It is defined by the Swedish Public Health authority as having struggles in running short distance, walking fast, or climbing stairs [4]. Due to lack of a unified definition for mobility disability across the globe, the burden between countries is difficult to compare [5].

The presence of mobility disability during early adulthood might lead to adverse consequences in the affected individuals. More specifically, young adults with acquired mobility limitations are more likely to increase their body weight [6] and follow an unhealthier lifestyle, which often includes low physical activity levels [7, 8], compared to their able-bodied peers. Lack of motivation, financial resources, and exercise expertise are commonly reported barriers for physical activity among people with mobility disability [9]. In addition, young adults with acquired mobility disability have a higher risk of lower social participation and health related quality of life (HRQoL) than those without mobility disability [10].

HRQoL constitutes a universal measurement for assessing self-perceived health covering several physical and mental health domains [11]. In the presence of a chronic condition, young adults are more likely to have lower HRQoL than middle-aged or older adults [12]. Moreover, adopting a healthy lifestyle during the life-course might have positive effects on HRQoL levels later in life [13]. Therefore, interventions including health promotion components might improve the HRQoL levels of young adults with mobility disability and prevent negative health consequences in the future.

Physical activity interventions are generally effective in improving HRQoL among adults [14–18]. Two comprehensive meta-analyses of physical activity interventions found that in healthy populations, being assigned to a physical activity group had positive effects on HRQoL compared to being assigned to a no-exercise control group [14, 15]. Similar effects have been found for physical activity interventions targeting old adults with mobility disability [16–18]. Nevertheless, an important component for the effectiveness of physical activity interventions in improving HRQoL, is the provision of supervised training [14, 15]. In fact, supervised training can provide social support [19] and motivation for health improvements [20]. However, given that people with mobility disability consider lack of financial resources as a barrier for physical activity [9], supervised training might be an unsustainable option for them.

Previous research suggests that physical activity interventions which target people with disabilities should consider the use of technological means [21]. Mobile apps could be an alternative to supervised training for young adults with mobility disability, as they are often inexpensive, incorporate self-monitoring and behavioral change techniques, and can easily reach large proportions of the population [22, 23].

Level of cardiorespiratory fitness is another more or less modifiable individual characteristic with known health benefits [24, 25], which could potentially influence HRQoL. More specifically, it is an indicator of habitual physical activity but it is also determined by a combination of individual and environmental factors [26]. Cross-sectional studies of healthy individuals have shown positive associations between cardiorespiratory fitness and HRQoL [27–30].

To the best of our knowledge, there is a lack of evidence regarding effective interventions in improving HRQoL in young adults with mobility disability. To date, the majority of interventions with this aim has been focused on older adults [31, 32]. Moreover, it is not known whether improved cardiorespiratory fitness is associated with better HRQoL in this population. Therefore, this study aims to examine potential differences in the effects of a mobile app versus a supervised health program on HRQoL and to determine the associations between cardiorespiratory fitness change and HRQoL in young adults with mobility disability.

## Methods

This is a secondary analysis of a parallel randomized controlled trial of two multicomponent interventions, with primary aim to compare the effects of a mobile app versus a supervised health program on physical activity levels in young adults with mobility disability [33]. A protocol with detailed information regarding the trial and the pre-defined outcomes has been previously published [33]. The follow up examinations took place at 6 weeks (midpoint of the intervention), 12 weeks (endpoint of the intervention), and 1 year after the start of the intervention (December 2019) according to the trial protocol [33]. The 12-week effects of the two interventions on the primary outcome (physical activity) and on two of the secondary outcomes (cardiorespiratory fitness and body composition) were compared in a previous study [34]. In brief, no significant differences were observed in the physical activity and cardiorespiratory fitness levels between the two groups, while waist circumference was significantly lower in the mobile app group [34]. The same study showed that cardiorespiratory fitness levels increased in both intervention groups [34]. That study was conducted before the 1 year follow-up was available and therefore included only the primary outcome and outcomes related to it [34].

### Study design

For the current study, the initial randomized controlled trial design was retained in order to compare the effects of the two interventions on HRQoL, which was among the pre-specified secondary outcomes of the trial (http://www.isrctn.com/ISRCTN22387524) [33], using an equivalence design. In addition, a longitudinal design with multiple cross-sectional waves was used merging all participants together, in order to study the association between changes in cardiorespiratory fitness and changes in HRQoL over 1 year. The decision to analyze randomized controlled trial data for fulfilling this aim is based on the previous observation that cardiorespiratory fitness levels improved in the study participants and on the availability of repeated measurements of both cardiorespiratory fitness and HRQoL. Since participation to the intervention could affect the association under study, analysis was also performed separately for each intervention group. The present study adheres to the CONSORT guidelines.

### Study participants

This study included young adults (18–45 years) that had acquired mobility disability over the past 3 years and had participated in the randomized controlled trial [33]. Individuals were considered as having mobility disability if they had self-reported mobility limitations in performing essential daily activities, such as getting dressed, or doing usual household and work tasks. Individuals with severe mobility limitations requiring mobility assistive devices or being unable to walk at a moderate to low intensity, were excluded [33]. Further inclusion criteria were having access to a smartphone and being able to understand and speak Swedish [33]. Recruitment took place in March 2018 and was performed by rehabilitation coordinators, at occupational, rehabilitation, and primary health care centers in the Stockholm region, Sweden [33].

### Randomization

During recruitment at the rehabilitation centers and before the first examination, staff from the TWITCH Health Capital in Stockholm randomly assigned participants to one of the two intervention groups. Participants were randomized to receive either a mobile app program (*n* = 55) or a supervised health program (*n* = 55) for 12 weeks [33]. In order to achieve an equal number of participants in the two intervention groups, a block randomization procedure was performed using a block size of two [33]. Randomization was performed using the SAS Proc Plan (SAS Institute Inc., Cary, NC, USA).

### Blinding

Due to the nature of the interventions, blinding of the participants was not possible. However, participants were not aware of their allocation group before the first examination. The investigators were blinded to the randomization and to the group allocation during the follow up assessments [33].

### Sample size

The sample size calculation of this study was based on moderate to vigorous physical activity (MVPA), which was the primary outcome of the randomized controlled trial [33]. For this population, a difference of 10 min/day in MVPA between the two intervention groups was desirable, and therefore this threshold was chosen for the sample size calculation [33]. The total number of participants needed to have this effect with 80% power, 5% of significance level with an expected 20% loss to follow up was 100 participants [33]. In order to prevent further loss in power from unexpected reasons, the decided sample size was 110 participants [33].

### The intervention groups

Both intervention groups had multiple components designed based on behavior change techniques, including the use of intrinsic motivation, self-monitoring, and goal setting, which have deemed to be effective in increasing physical activity levels in adults [35].

#### Mobile app program

The mobile app program combined the use of three mobile apps, including a widely used walking app called Acupedo, a home-based body weight based training app, and a food photography app named LogMyFood [33]. The training app was created by the Swedish Military and consists of several visually explained body exercises [36]. Participants were advised to use it at least three times per week, choosing the level and the type of exercises they preferred [33]. This group also received three face-to-face group consultations which took place at baseline for introducing the apps, at the midpoint of the intervention to encourage adherence and goal setting, and at the endpoint of the intervention aiming to increase their motivation to be physically active [33].

#### Supervised health program

In the supervised group, participants received health coaching and an individualized weekly personal training session for 1 h [33]. They were also asked to use the LogMyFood food photography app and were encouraged to exercise by themselves at least twice per week and walk daily for at least 30 min [33]. In contrast with the mobile app group, participants in the supervised group could share food intake information through the LogMyFood app [33]. The supervised components where designed according to the transtheoretical model and the social cognitive theory [37]. The personal training sessions were taking place at fitness centers in Stockholm and were combining aerobic workout and strength exercises that were designed to be easily adoptable to the home environment [33]. During these sessions, participants were also receiving a 5–10 min motivational interviewing and feedback by their trainer [33]. In total, six experienced trainers employed by the fitness centers delivered the personal training sessions and each participant was assigned to one of them during the whole intervention period. All personal trainers had 2–3 group meetings during the intervention, in order to discuss their training practices. Participants in this group also attended three personal meetings with a health educator, aiming to promote better physical activity and dietary habits [33].

### Measurements

#### Outcome

Health related quality of life (HRQoL) was assessed at baseline and at the follow up examinations with the SF-36 health survey which is a widely used instrument [38]. It consists of 36 questions that cover eight domains of HRQoL: physical functioning, role limitations due to physical health problems, bodily pain, general health, vitality, social functioning, role limitations due to emotional problems, and mental health [39]. The score of each domain is ranging from 0 to 100, with higher scores indicating better HRQoL [39]. In addition, the SF-36 domain scores can be collapsed to generate the physical component summary and the mental component summary scores, ranging between 0 and 100 [39].

#### Exposure

Cardiorespiratory fitness was estimated at all examination points from the results of the Ekblom-Bak test, which is a submaximal cycle ergometer test that estimates V˙O_2_max in ml/kg/min using sex specific equations that account for age [40]. V˙O_2_max is an objective measure of cardiorespiratory fitness and represents the maximum volume of oxygen that the body can utilize per unit of time during intensive exercise [41].

#### Demographic and clinical characteristics (age, sex, chronic conditions)

Demographic and clinical information including age, sex, and chronic conditions was collected at baseline through a self-reported questionnaire [33]. Due to the narrow age range of participants, age was analyzed as continuous variable. Regarding the presence of chronic conditions, participants were asked one Yes/No question. Namely, they were asked whether they had any long-term illness, complications due to accident, impaired function, or long-term health problems.

#### Smoking

Smoking status was assessed at baseline through three Yes/No questions including whether they were daily smokers, occasional smokers, and if they ever used to smoke daily for at least 6 months [33]. Participants answering “No” to all three questions were categorized as “Never smokers”, those who answered “No” on whether they were daily smokers or occasional smokers but answered “Yes” on if they ever used to smoke daily for at least 6 months, were categorized as “Previous smokers”. Participants answering “Yes” on being daily or occasional smokers, were categorized as “Smokers”.

#### Body mass index (BMI)

Body mass index (BMI) was calculated as body weight in kg divided by the square of height in meters. Height and weight were measured without shoes and were rounded to the nearest 0.5 cm and 0.1 kg respectively, using validated scales [33].

#### Moderate to vigorous physical activity

Moderate to vigorous physical activity (MVPA) was measured at each examination point with the use of accelerometers (Actigraph GT3X+) for seven consecutive days [33]. In order to consider the measurements as valid, participants had to wear the device on their waist for at least 10 h per day for a minimum of three weekdays and one weekend day [33].

### Statistical analyses

Statistical analyses were performed using Stata/IC 15. A significance level of < 0.05 was set for all statistical tests. Baseline characteristics of all participants and within each intervention group are presented in Table 1 as mean ± standard deviation (SD) for the continuous variables and as frequencies and percentages for the categorical variables. In order to test for mean changes in V˙O_2_max and the SF-36 domain scores between baseline and each follow up examination, two tailed t-tests were applied. The results are presented as mean changes ± SD between baseline and each follow up examination.

**Table 1:** Baseline characteristics of total participants and stratified by intervention group, *N* = 110

Baseline characteristics	Total (***N*** = 110)	Mobile app program (***n*** = 55)	Supervised program (***n*** = 55)
**Females, N (%)**	90 (82.6)	47 (85.5)	43 (78.2)
**Age (years),** mean ± SD	35.1 ± 6.4	35.6 ± 6.2	34.5 ± 6.5
**BMI (kg/m**^**2**^**),** mean ± SD	26.7 ± 5.5	26.3 ± 5.7	27.2 ± 5.2
**Smokers, N (%)**			
Never	61 (57)	27 (51)	34 (64)
Previous	24 (23)	12 (23)	12 (23)
Occasional	16 (15)	11 (21)	5 (9)
Daily	5 (5)	3 (5)	2 (4)
**Chronic condition(s), N (%)**	95 (88)	47 (87)	48 (89)
**MVPA (min/day),** mean ± SD	44.34 ± 22.2	48.4 ± 23.3	40.3 ± 20.6
**V˙O**_**2**_**max (ml/kg/min),** mean ± SD	35.6 ± 8.3	36.1 ± 7.8	35.1 ± 8.8
**SF-36 domains (score),** mean ± SD			
Physical functioning	73.0 ± 16.5	73.0 ± 19.2	72.9 ± 13.4
Physical role functioning	65.5 ± 25.8	66.0 ± 27.7	64.9 ± 24.1
Bodily pain	50.7 ± 19.6	50.6 ± 21.4	50.8 ± 17.9
General health	52.0 ± 21.6	50.5 ± 21.2	53.5 ± 22.1
Vitality	38.9 ± 18.0	38.3 ± 8.3	39.6 ± 17.7
Social functioning	66.3 ± 27.4	63.4 ± 28.3	69.2 ± 26.3
Emotional role functioning	73.2 ± 25.2	75.2 ± 22.7	71.3 ± 27.5
Mental health	53.8 ± 15.7	51.7 ± 15.3	55.9 ± 16.0
Physical component summary	44.5 ± 8.9	44.5 ± 9.6	44.5 ± 8.2
Mental component summary	39.8 ± 8.1	39.1 ± 7.3	40.4 ± 8.8

Abbreviations: *SD* Standard deviation; *BMI* Body mass index, *MVPA* Moderate to vigorous physical activity

The effects of the mobile app program compared to the supervised health program on the SF-36 scores were examined using intention-to-treat analysis. Between group differences in the mean changes of SF-36 scores from baseline to midpoint, endpoint, and 1-year were estimated using simple linear regression models, with the supervised group chosen as referent.

Mixed-effects linear regression models were fitted for estimating the association of changes in V˙O_2_max at midpoint, endpoint, and 1 year from baseline, with changes in the SF-36 scores. Mixed-effects linear regression is an extension of the generalized linear model and was suitable for this analysis, as it accounts for correlations between repeated measurements within participants and can model both mixed and random effects [42]. The intraclass correlation coefficient (ICC) was calculated for each model. All models were adjusted for age, sex, BMI, chronic conditions, and smoking, since they are predictors of both cardiorespiratory fitness [26] and HRQoL [43]. In addition, separate models were fitted for each intervention group, in order to examine possible effect modification of the intervention group on the relationship between changes in V˙O_2_max and changes in the SF-36 scores.

Sensitivity analysis was also performed for comparing baseline characteristics between those who attended the 1-year follow up and those who did not. In addition, changes in V˙O_2_max, MVPA and SF-36 scores from the midpoint to the endpoint of the intervention, were compared between those who attended the 1-year follow up and those who did not. Two-tailed t-tests and chi-squared tests were used for these comparisons with level of significance α = 0.05.

## Results

**Fig. 1:**
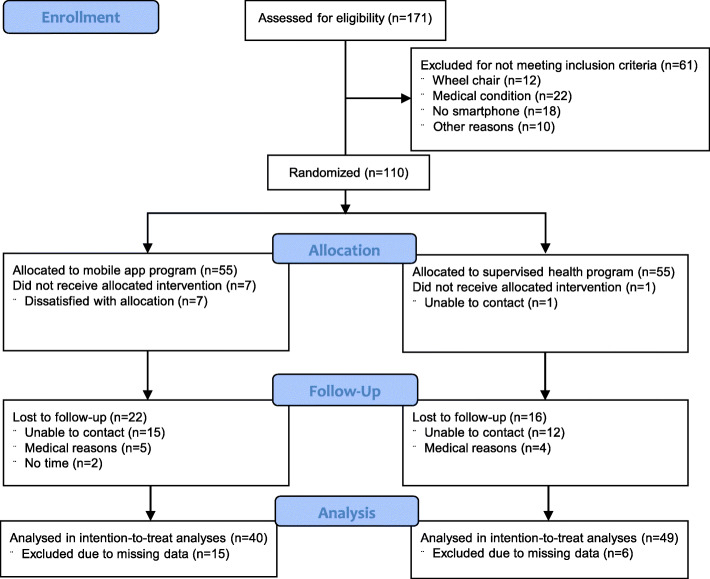
Participant flow chart

The flow-chart of study participants is presented in Fig. 1. In total, 110 individuals were included in the analysis and 58% of them attended the 1-year follow up. Among those that were lost to follow up, 5 participants in the mobile app group and 4 in the supervised group withdrew from the study due to medical reasons. However, none of these reasons was related to participation to the study. Baseline characteristics are presented in Table 1. Most participants were females (82.6%) and mean age was 35.1 (SD = 6.4) years. Results from the SF-36 questionnaire indicated that the average score in the physical component summary was 44.5 (SD = 8.9) and in the mental component summary was 39.8 (SD = 8.1). The highest domain scores were in emotional role functioning 73.2 (SD = 25.2) and physical functioning 73.0 (SD = 16.5), while the lowest score was in vitality 38.9 (SD = 18.0).

### Effect of mobile app versus supervised training on the SF-36 domains

**Fig. 2:**
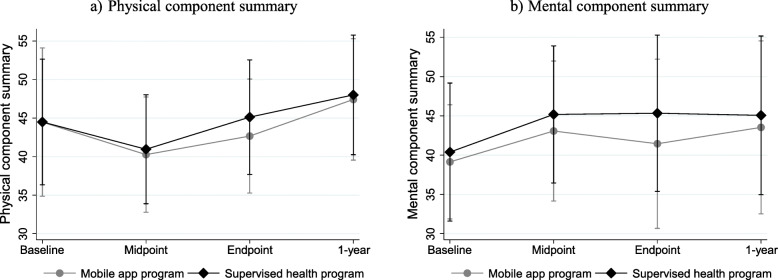
Mean component summary scores of SF-36 ± SD during follow up for each intervention group

In total, 40/55 participants from the mobile app and 49/55 from the supervised health program were included in the intention-to-treat analysis, as they had completed the SF-36 questionnaire in at least one follow-up examination. Between the two intervention groups there were not significant differences in the mean changes of SF-36 scores from baseline to each follow up. (see Table 2). Comparing the mobile app group to the supervised group at the endpoint, the difference in the mean change of the physical and mental component summary was − 3.06 (95% CI: − 7.81 to 1.68) and − 3.62 (95% CI: − 8.96 to 1.72) respectively. At the 1-year follow up these differences were 0.09 (95% CI: − 6.27 to 6.46) for the physical component summary and 0.57 (95% CI: − 5.58 to 6.72) for the mental component summary. Figure 2 illustrates the mean scores ± SD of the physical and mental component summary of SF-36 at each examination point for each intervention group.

**Table 2:** Between group differences in the mean changes of the SF-36 scores from baseline to each follow-up

	Δ (Baseline to Midpoint)	Δ (Baseline to Endpoint)	Δ (Baseline to 1 year)
**SF-36 domains (score)**	**β coefficient (95% CI)**^a^	**β coefficient (95% CI)**^a^	**β coefficient (95% CI)**^a^
Physical functioning	−1.49 (− 9.05, 6.06)	−7.12 (− 15.34, 1.10)	−1.93 (− 12.16, 8.29)
Physical role functioning	−7.66 (− 24.04, 8.73)	- 13.93 (− 30.33, 2.48)	0.35 (− 21.09, 21.79)
Bodily pain	−1.45 (− 15.02, 12.12)	−6.94 (− 19.75, 5.86)	2.16 (− 13.16, 17.49)
General health	−0.33 (− 14.80, 14.15)	−7.69 (− 21.13, 5.74)	2.64 (− 14.12, 19.40)
Vitality	−2.65 (− 13.73, 8.42)	−8.85 (− 21.20, 3.49)	−1.01 (− 15.37, 13.35)
Social functioning	−1.23 (− 17.16, 14.69)	−3.81 (− 9.80, 17.41)	2.05 (− 15.20, 19.31)
Emotional role functioning	−14.68 (− 29.61, 0.26)	- 16. 01 (− 32.46, 0.45)	− 7.71 (− 26.07, 10.65)
Mental health	4.41 (− 4.33, 13.15)	− 5.44 (− 15.65, 4.77)	7.11 (− 4.22, 18.43)
Physical component summary	−0.82 (− 5.81, 4.17)	−3.06 (− 7.81, 1.68)	0.09 (− 6.27, 6.46)
Mental component summary	−1.65 (− 6.65, 3.36)	−3.62 (− 8.96, 1.72)	0.57 (− 5.58, 6.72)

^a^ β coefficients of the linear regression models in intention-to-treat analysis with supervised health program as reference, *n* = 89; Abbreviations: *CI* Confidence intervals

### Changes in V˙O_2_max and SF-36 domains

Mean changes in V˙O_2_max levels and the SF-36 domain scores between baseline and each follow up examination are presented in Table 3. V˙O_2_max was significantly higher at the follow up examinations compared to baseline. In addition, the scores of physical functioning, bodily pain, mental health, and mental component summary were improved at 1 year from baseline. Physical role functioning, general health, and vitality scores were improved during the intervention, but did not remain at the same level in the 1-year follow up. In addition, physical functioning, social functioning, and physical component summary scores decreased during the intervention but reached the baseline levels at the 1-year follow up.

**Table 3:** Mean changes in V˙O_2_max and SF-36 scores ± SD between baseline and follow up examinations, *n* = 89

Variables	Δ (Baseline to Midpoint)	***p***-value^a^	Δ (Baseline to Endpoint)	***p***-value^a^	Δ (Baseline to 1 year)	***p***-value^a^
**V˙O**_**2**_**max (ml/kg/min)**	1.61 ± 2.7	< 0.001	2.0 ± 2.9	< 0.001	1.24 ± 3.7	0.016
**SF-36 domains (score)**						
Physical functioning	−29.7 ± 17.8	< 0.001	− 10.4 ± 19.3	< 0.001	9.0 ± 19.7	< 0.001
Physical role functioning	9.1 ± 38.7	0.03	6.0 ± 38.6	0.148	7.5 ± 41.3	0.152
Bodily pain	− 3.1 ± 31.9	0.361	7.3 ± 29.8	0.024	8.3 ± 29.5	0.03
General health	8.9 ± 34.0	0.016	9.5 ± 31.3	0.006	7.2 ± 32.3	0.082
Vitality	6.5 ± 26.0	0.022	5.3 ± 28.9	0.09	4.9 ± 27.7	0.168
Social functioning	5.1 ± 37.4	0.205	−9.1 ± 31.5	0.009	4.8 ± 33.3	0.260
Emotional role functioning	2.2 ± 35.8	0.555	1.3 ± 39.9	0.749	2.0 ± 35.6	0.659
Mental health	0.2 ± 20.6	0.929	12.9 ± 23.8	< 0.001	13.3 ± 22.1	< 0.001
Physical component summary	−3.6 ± 11.7	0.005	− 0.54 ± 11.1	0.653	2.8 ± 12.3	0.072
Mental component summary	4.4 ± 11.8	0.001	3.7 ± 12.5	0.007	3.5 ± 11.8	0.021

^a^ *p*-values from t-tests

### Association of changes in V˙O_2_max with the SF-36 domains

The mixed-effects linear regression models indicated that changes in V˙O_2_max at midpoint, endpoint, and 1 year from baseline, were positively associated with changes in the physical role functioning (β = 1.06, 95% CI: 0.11 to 2.01, ICC = 0.86), general health (β = 1.37, 95% CI: 0.56 to 2.19, ICC = 0.86), vitality (β = 0.83, 95% CI: 0.02 to 1.64, ICC = 0.80), and emotional role functioning (β = 1.34, 95% CI: 0.27 to 2.42, ICC = 0.79) domains of SF-36 (see Table 4). After adjusting all models for possible confounders (age, sex, BMI, chronic conditions, and smoking), only the associations of changes in V˙O_2_max with changes in general health (β = 1.30, 95% CI: 0.48 to 2.13, ICC = 0.86) and emotional role functioning (β = 1.18, 95% CI: 0.11 to 2.25, ICC = 0.78) remained statistically significant. When measuring these associations within each intervention group, changes in V˙O_2_max were significantly associated with changes in the SF-36 scores only in the supervised health program. More specifically, both crude and adjusted models in the supervised group indicated associations between changes in V˙O_2_max and changes in the general health (adjusted model β = 1.88, 95% CI: 0.87 to 2.90, ICC = 0.87) and emotional role functioning (adjusted model β = 1.37, 95% CI: 0.18 to 2.57, ICC = 0.85) domains.

**Table 4:** Association of changes in V˙O_2_max with changes in the SF-36 scores from baseline to each follow up

	Total participants		Mobile app program		Supervised program	
	**β coefficient (95% CI)**^b^		**β coefficient (95% CI)**^b^		**β coefficient (95% CI)**^b^	
**Δ (SF-36 domains)**	**Crude**	**Adjusted**^a^	**Crude**	**Adjusted**^a^	**Crude**	**Adjusted**^a^
Physical functioning	0.24 (− 0.83, 1.31)	0.13 (− 0.95, 1.21)	− 0.54 (− 2.27, 1.19)	−0.46 (− 2.23, 1.30)	0.65 (− 0.64, 1.95)	0.33 (− 1.02, 1.69)
Physical role functioning	1.06 (0.11, 2.01)	0.94 (− 0.01, 1.90)	1.32 (− 0.22, 2.86)	0.99 (− 0.56, 2.53)	0.92 (− 0.29, 2.13)	0.86 (− 0.37, 2.09)
Bodily pain	0.24 (− 0.87, 1.36)	− 0.01 (− 1.11, 1.09)	0.49 (− 1.20, 2.17)	0.10 (− 1.54, 1.74)	0.11 (− 1.38, 1.60)	− 0.07 (− 1.59, 1.44)
General health	1.37 (0.56, 2.19)	1.30 (0.48, 2.13)	0.49 (− 0.87, 1.86)	0.19 (− 1.18, 1.56)	1.93 (0.93, 2.93)	1.88 (0.87, 2.90)
Vitality	0.83 (0.02, 1.64)	0.73 (− 0.06, 1.53)	1.29 (− 0.13, 2.71)	0.92 (− 0.47, 2.32)	0.55 (− 0.41, 1.51)	0.60 (− 0.37, 1.57)
Social functioning	− 0.58 (− 1.87, 0.71)	−0.77 (− 2.07, 0.53)	−0.63 (− 2.72, 1.46)	− 1.02 (− 3.13, 1.09)	−0.58 (− 2.24, 1.07)	−0.80 (− 2.49, 0.90)
Emotional role functioning	1.34 (0.27, 2.42)	1.18 (0.11, 2.25)	1.04 (−0.91, 3.0)	0.80 (− 1.14, 2.74)	1.45 (0.27, 2.63)	1.37 (0.18, 2.57)
Mental health	0.24 (−0.67, 1.16)	0.28 (−0.62, 1.18)	0.38 (−1.41, 1.49)	− 0.26 (− 1.62, 1.10)	0.37 (− 0.81, 1.54)	0.53 (− 0.69, 1.76)
Physical component summary	0.23 (− 0.13, 0.59)	0.19 (− 0.17, 0.55)	0.09 (− 0.48, 0.65)	0.02 (− 0.55, 0.59)	0.31 (−0.15, 0.78)	0.26 (− 0.21, 0.74)
Mental component summary	0.24 (−0.18, 0.66)	0.19 (− 0.22, 0.59)	0.28 (− 0.49, 1.04)	0.10 (− 0.62, 0.81)	0.22 (− 0.25, 0.69)	0.22 (− 0.25, 0.70)

^a^ Adjusted for age, sex, BMI, chronic conditions, smoking; ^b^ β coefficients of the mixed effects linear regression models, *N* = 110; Abbreviations: *CI* Confidence intervals

### Sensitivity analyses

Sensitivity analyses indicated that participants who did not attend the 1-year follow up did not differ from the participants who attended the 1-year follow up regarding the baseline characteristics. Among the participants that did not attend the 1-year follow-up, 65% were from the mobile app group. In the mobile app group, 7 participants were dissatisfied with the allocation and dropped out before receiving the intervention. During the follow up, the attrition rates were similar in both groups. In addition, those who did not attend the 1-year follow up but had attended the endpoint follow up, had smaller increases in their V˙O_2_max from midpoint to endpoint, compared to those who attended the last follow up (0.9 vs 2.4, *p* = 0.03).

## Discussion

This study found no significant differences in the mean change of HRQoL scores from baseline to each follow up between a mobile app and a supervised health program delivered to young adults with mobility disability. Positive associations were observed between changes in cardiorespiratory fitness over 1 year and changes in the physical role functioning, general health, vitality, and emotional role functioning domains of HRQoL. After adjusting for potential confounders, the observed associations with physical role functioning and vitality did not remain statistically significant. Moreover, stratification by intervention group resulted in associations between changes in cardiorespiratory fitness and general health and emotional role functioning, only in the supervised health program.

### Comparison with previous studies

A small number of previous studies focused on clinical populations has evaluated the effects of mobile app training on HRQoL [44–47]. Among those studies, two found that receiving mobile app training had higher effects on HRQoL compared to receiving usual care which included unsupervised self-rehabilitation [44] or one home visit by a physician who provided nutrition and exercise advice [45]. Another study found similar effects between a mobile app program and the provision of an exercise brochure [46]. However, the post-intervention scores were higher compared to baseline in both groups [46]. Similarly, another study without control group found higher post-intervention scores at follow up compared to baseline [47]. Overall, the available evidence indicates that mobile app interventions might improve HRQoL in clinical populations. In the current study, the effects of the mobile app program were compared to a supervised health program which according to literature was expected to improve HRQoL [15, 48, 49]. Furthermore, the attrition rate within the mobile app group in our study, especially after the allocation, is higher compared to other studies of mobile app training [50]. This could be explained by the fact that previous studies did not have supervised training as a comparator, but instead provision of advice or no intervention, which might had been seen less attractive than using a mobile app [50]. However, the total attrition rates of this study are similar to previous studies of mobile apps [50].

Associations between cardiorespiratory fitness and specific domains of HRQoL have been previously observed in cross-sectional studies, however showing partially inconsistent results. More specifically, a study of healthy young males that looked into the different domains of HRQoL found positive associations only with the general health domain [27], whereas two other studies in a similar population that assessed the component summary scores of HRQoL found positive associations with both physical and mental health [28, 29]. A study of postmenopausal women in which all domain and component summary scores of SF-36 were assessed, found that cardiorespiratory fitness was associated only with the mental component summary [30]. In contrast, a study of two national representative samples in Sweden that assessed self-perceived physical and general health with two questions, found that cardiorespiratory fitness was a predictor of physical health [51]. The discrepancies in specific domains between these studies could be explained by the different components of HRQoL that were assessed and the different sexes of the participants. Nevertheless, the results of the current study might differ from previous studies for several reasons. First, in contrast to previous studies, this study looked at the associations between changes in cardiorespiratory fitness and HRQoL. In addition, the focus was on people with mobility limitations who had received physical activity interventions, a factor that seemed to modify the association of cardiorespiratory fitness changes with HRQoL.

This study also supports previous evidence regarding the possible benefits of supervised training on HRQoL. Two studies of older adults with mobility limitations reported higher increases in general health [48] and emotional role functioning [49] respectively, in those receiving supervised training compared to unsupervised training at home. Moreover, in a meta-analysis studying the effects of physical activity interventions on HRQoL, it was shown that interventions including supervised training were more effective compared to educational-motivational interventions [15].

### Strengths and limitations

This study has several strengths. Most importantly, to the best of our knowledge this is the first study to compare the effects of mobile app training to supervised training on HRQoL as well as to examine the association of changes in cardiorespiratory fitness with HRQoL, in people with mobility disability. In addition, the methods chosen had several benefits. Most importantly, the randomized controlled trial study design using intention to treat analysis, was allowing to compare the effects of the two interventions on HRQoL with a very low risk for confounding as all the characteristics of the participants were randomized in each group. Moreover, studying the population longitudinally allowed us to assess whether changes in cardiorespiratory fitness were associated with changes in HRQoL on an individual level over 1-year period, accounting for possible correlations between the repeated measurements with the use of mixed effects models. Finally, cardiorespiratory fitness was estimated with a submaximal cycle ergometer test, minimizing the risk of exposure misclassification, while HRQoL was measured with a widely used questionnaire [38], which allowed comparability with previous studies.

Nevertheless, there are also some limitations that need to be considered. A threat for the internal validity of this study is the high attrition rates. Especially when comparing the effects of the two interventions on HRQoL, it is likely that the estimates have been affected by selection bias. As the sensitivity analysis showed, higher proportion of the dropouts was from the mobile app group. Although there were no differences regarding changes in HRQoL from midpoint to the endpoint between those who attended the 1-year follow up and those who did not, it is possible that participants who did not attend the midpoint or endpoint follow up had small or negative changes. This might have led to the underestimation of the differences in the mean change of HRQoL scores between the two groups, having the supervised group as reference. Regarding the estimates of the association of changes in cardiorespiratory fitness with HRQoL, mixed effects regression models use the maximum likelihood method to handle missing data by estimating the mean values from the available data at each time point [52]. However, if the missing values are related to both HRQoL and cardiorespiratory fitness, these estimates might have been biased [52]. Since it is likely that those with lower HRQoL and cardiorespiratory fitness were lost to follow up, the estimated associations might have been diluted.

The generalizability of this study might have also been limited due to the high attrition rates and the exclusion criteria. Certain characteristics of the study population such as higher proportion of females than males, were representative of young adults with mobility disability living in Stockholm [3, 4]. However, the participants that were lost to follow up might have been different than those who remained to the study. These differences might have been related to the severity of mobility disability during the follow-up, possibly leading to higher retention rates among those with less severe symptoms. In addition, exclusion of people without access to a smartphone or that were not speaking Swedish during the enrollment, further restricts the generalizability of this study. Moreover, people enrolled in this study were recruited from rehabilitation centers, excluding individuals that did not seek care. Therefore, the results of this study could possibly be generalized to young adults with moderate mobility disability living in Stockholm or in other cities in Sweden, who would seek care in rehabilitation centers.

Further limitations of this study lay on the interpretation of the results. More specifically the sample size of the study was probably not adequate to detect differences in the effects of the mobile app compared to the supervised program on HRQoL, since it was calculated based on the primary outcome. However, equivalence of these effects cannot be implied, as such inference would require a larger sample size [53]. Furthermore, the nature of the data does not allow the inference of causal effects of cardiorespiratory fitness on HRQoL. Although according to theory this relationship could be very likely [54], the possibility of inverse causality cannot be excluded. Therefore, it could also be possible that HRQoL affects cardiorespiratory fitness.

### Future studies

More evidence is needed regarding the effects of mobile app training on HRQoL in adults with mobility disability. Future research could include larger samples and compare these effects not only with supervised training but also with usual treatment. In addition, further studies in young adults with mobility disability are needed in order to replicate the associations between changes in cardiorespiratory fitness and HRQoL, to investigate the existence of a causal relationship, and to better understand the role of supervised training in this association.

## Conclusions

This study found positive associations between cardiorespiratory fitness and the general health and emotional role functioning domains of HRQoL in young adults with mobility disability who received supervised training. It remains unclear whether mobile app training has similar effects with supervised health programs on HRQoL in people with mobility disability. Further studies are needed to better understand these effects.

## Data Availability

The datasets generated and analyzed during the current study are not publicly available but are available from the corresponding author on reasonable request.

## Abbreviations

BMI: Body mass index
CI: Confidence interval
HRQoL: Health-related quality of life
ICC: Intraclass correlation coefficient
ICF: International classification of functioning, disability and health
MVPA: Moderate to vigorous physical activity
SD: Standard deviation
SF-36: Short form (36) health survey
WHO: World health organization
